# Developing a core competency framework for advanced practice nursing in mainland China: a sequential exploratory study

**DOI:** 10.1186/s12912-023-01335-4

**Published:** 2023-05-23

**Authors:** Hongxia Guo, Wei Zhu, Jiping Li

**Affiliations:** 1 Department of Nursing, West China Hospital, Sichuan University/West China School of Nursing, Sichuan University, Chengdu, Sichuan China; 2 Nursing Department of West China Hospital, Sichuan University, Chengdu, Sichuan China

**Keywords:** Advanced practice nursing, Advanced practice nurse, Competency framework, Professional role, Sequential exploratory study

## Abstract

**Background:**

Advanced Practice Nursing (APN) have been highly valued and an integral part of the health care system. Development and establishment of new APN roles is a complex process that has resulted from a wide variety of reasons, key component is a lack of a competency map delineation and role evaluation. Currently, however, competence framework has not been compared at an international level. In mainland China, APN have been introduced in some organizations but their competency domains have not yet been clearly defined, this study aimed to identify the core competencies for advanced practice nursing.

**Methods:**

This study was performed in two phases: first, in-depth and semi-structured individual interviews with 46 participants from key stakeholders were carried out followed by a qualitative content analysis, then an item pool of core competencies was constructed by extracting data from the first phase and the results from previous studies, scales and documents; second, a Delphi technique was conducted with the participation of 28 experts from 7 areas of China to form the final core competency framework for advanced practice nursing.

**Results:**

Through the qualitative phase, the core competency framework with six domains and 70 items emerged and then entered into the Delphi phase. Twenty-eight of 30 experts finished 2 rounds of Delphi approaches. The final core competencies for advanced practice nursing consisted of six domains with 61 items, including direct clinical nursing practice, research and evidence-based nursing practice, professional development, organization and management, mentoring and consultation, and ethical/legal practice.

**Conclusion:**

This core competency framework consisted of six domains with 61 items can be used in competency-based education to cultivate advanced practice nurses as well as competency level assessment.

**Supplementary Information:**

The online version contains supplementary material available at 10.1186/s12912-023-01335-4.

## Introduction

With the increasing need for improved nursing services and outcomes, APN has spread and accepted worldwide and was proven to have advantages in improving access to care, decreasing waiting time, and cost containment of health care [1]. To date, approximately 70% of nations have some form of advanced practice nursing [2]. APN is the expanded healthcare services and interventions provided by nurses with an advanced capacity [3]. The advanced practice nurse is granted to a nurse who has completed additional education to acquire expert knowledge, complex decision-making skills, and the clinical competencies to provide expanded healthcare services within the context of nursing [4]. Identifying the definition of nursing competencies is the foundation of the conceptualization and development of advanced practice nursing roles [5, 6]. Nursing competency is a mastery of knowledge, clinical, interpersonal and technical skills, which is the basis for good performance in the clinical environment [7]. In 1996, Hamric had put forward core competencies for advanced practice nurses(APNs), which included seven domains that took clinical practice, nursing research, health coaching and education, collaboration, ethical decision making, and leadership into consideration [8]. In 2011, the American Association of Colleges of Nursing (AACN) designed six core competencies for master’s education in nursing, such as changes for quality care, culture of excellence, and translating evidence into practice [9]. In 2010, the Canadian Nurses Association published the core competency framework for nurse practitioners: clinical practice, collaboration, consultation and referral, research, and leadership [10]. In Australia, the Australian Nursing & Midwifery Council (ANMC) included three core competencies for nurse practitioners: dynamic practice, professional efficacy and clinical leadership [11]. In the UK, the Royal College of Nursing (RCN) established the core competency framework for APN [12].

Hong Kong is an area with early development of advanced practice nurses in China, the nurse specialist role was first piloted in 1993 and an advanced practice nursing role piloted in 2003. Nurse practitioner (NP) is the title of advanced practice nurse in Taiwan, who not only have a master's degree or doctor's degree, but also are registered nurses who have received the master's level education of nurse practitioner. Chinese mainland has established certified registered nurse and certified nurse systems to cultivate nurses with different competency levels under accompanying training and certification regulations. Although the current regulations for nurses, launched in 2008, did not have any APN roles or APN-related descriptions, Chinese researchers began to explore the localization of the role of advanced practice nurse based on the foreign concept model, and found that training advanced practice nurse from master level is the trend of the development of nursing specialization in recent years. By the end of March 2015, there were 84 clinical master's programs in nursing in China [13]. This resulted in a significant increase in the number of clinical nurses with master's degrees, which prompted China to establish an advanced practice specialty nursing program to prepare nurses for future work with medical specialists. Review of the literature revealed that advanced practice nurse role with higher consensus in china were clinical practitioner, manager, educator, researcher, coordinator and consultant [14]. This result is consistent with results of the international reasearech [15], but competences required for APN are wide heterogeneity, with a total of 16 was mentioned in the literature [14]. Clinical practice ability, research ability and management ability are higher consensus in the process of developing advanced practice in specialty areas, there is much confusion between Specialist Nurses(SN) and advanced practice nurses. Specialist nurses are probably those who can deal with situations in a specific area of practice. Advanced practice nurses not only perform proficiently and possess specialized knowledge, but also have original thinking, innovation and the ability to analyse complex situations in delivering services and leading changes [13]. APNs are at the frontier of their field and at the top of the clinical ladder [16]. The roles of Specialist Nurses(SN) have been defined and regulated in some organizations of mainland china. Some researchers have explored the core competencies of advanced practice nurses in different specialized fields, such as gastroenterology nursing specialists [6], oncology advanced practice nurses [17], critical care advanced practice nurses [18], and advanced midwifery practitioners [19]. In addition, previous studies in China did not provide a clear definition of specialist nurse or advanced practice nurse. The core competencies for APN role are not clearly defined, with confusion regarding the APN title more prevalent since delineation of the Specialized Nurse roles. Some intuitive or logical methods of categorization were used, with small convenience samples, resulting in validity and reliability. Under this condition, we aimed to identify a competency framework for APNs through sequential exploratory research.

The definition of advanced practice nurses(APN) used in our study is defined as a registered nurse with a master's degree or above and with at least 5 years of working experience who has received academic training and practical training, acquired the expert knowledge base, complex decision-making skills and clinical competencies for expanded practice.

## Methods

An exploratory sequential study was designed. We conducted a two-phase approach to develop the core competencies for advanced practice nursing: (1) Phase 1: a literature review and qualitative interviews were conducted to draft the initial competency framework; Phase2: The Delphi technique was used to identify the framework.

### Phase 1: Semi-structured interviews with Key Stakeholders

This phase adopted a qualitative descriptive study to explore how APN’s core competencies are performed in clinical practice. An open-ended interview outline was constructed under the guidance of Hamric’s core competency framework.

### Participants

This step involves identifying key stakeholders who would affect or be affected when the model of care changed and introduced an APN role. In our research, a mixed sampling strategy combining the purpose and snowball sampling method was used to recruit participants, including nursing managers, educators, experts from hospitals, government departments or associations related to health care, nursing education, and nurses with master of nursing specialist degrees. The inclusion criteria for nursing educators were as follows: (1) served as a supervisor of graduate nursing students and (2) had the title of associate professor above. The inclusion criteria for nursing managers from the hospital were as follows: (1) ever had postgraduate nursing employees; (2) served as a nursing manager for at least 1 year; (3) had clinical experience for 5 years or above; and (4) had a middle-level professional title or above. The inclusion criterion for experts from government departments or associations was to be familiar with nursing management and education policy in China. The inclusion criterion for nurses was having clinical experience of at least five years. A total of 46 participants volunteered to participate in face-to-face, semi-structured, and open-ended interviews. An interview framework was predefined to guide the interviews (Table 1).

**Table 1:** Framework for the interviews

Participants	Questions
Nursing experts, nursing managers, experts from government or associations related to nursing education	Q1: What do you think should be included in the job responsibilities of APN engaged in clinical practice? What kind of work do you expect them to do? Q2: What do you think should be the abilities of APNs? What are the specific aspects of these capabilities? Q3: Please give some suggestions for the development of the core competences of APN in China
Nurses with master of nursing specialist degrees	Q1: What do you think should be the abilities of APN? What are the specific aspects of these capabilities? Q2: Please talk about a time that you think is a successful work experience, and why do you think it is success? What do you learn from this? Q3: Please talk about a time that you think is a failed work experience, and why do you think it is failure? What do you learn from this? Q4: What factors do you think affect your ability level? Q5: Do you think you can become an advanced practice nurse after graduation? If so, what do you think you need to prepare?

Between March and September 2017, qualitative interviews were carried out at the study participants’ workplaces and/ or place of study. Forty-six in-depth individual interviews were conducted by the researcher, with the interview time ranging from one to two hours. The researcher had no relationships with these participants and promised the confidentiality of the interviews again before the interviews. During the interviews, the researcher listened to the participants and took notes of their tones, expressions, and body language to collect nonverbal information. At the end of the interviews, the participants were asked to verify the accuracy of the information. Each interview was audiotaped and then transcribed within 24 h after the interview.

### Ethical considerations

This study was approved by the ethics committee of West China Hospital, Sichuan University (ID: 2017–177). Before starting the interviews, the researcher explained the purpose and procedure of the interviews in a private and quiet room and got the written informed consents from participants.

### Data analysis

Conventional content analysis was adopted to analyse the transcripts through reading, coding, and thematising [20].

### Trustworthiness

Some strategies were adopted to ensure the trustworthiness of this qualitative research process. Regarding credibility, the interview questions were designed to collect information that reflected the real experiences of the participants, and the researcher paraphrased all dialogues to ensure accurate interpretation of the participants’ opinions. Peer checking between two researchers was used to enhance the confirmability of this study. Regarding dependability, audit trials were taken by collecting, thematising and analysing data [21].

### Phase 2: Delphi Process

In phase 2, for each initial competency domain, competencies relevant to each domain were drafted by combining the results of the literature review and interviews. Up to 83 competencies were formed at this stage. Then, five experts reviewed and refined the competencies by identifying redundancies, integrating related concepts, and removing competencies with a clinical focus interactively. The experts’ evaluation used the 4-point rating scale (1 = not relevant, 2 = somewhat relevant, 3 = quite relevant, and 4 = very relevant). The items were deleted when three or more experts gave 1 or 2, indicating redundancy with other items in the same category. Totally, 13 items were deleted.

The Delphi technique was used to identify competency domain delineation of the framework for APN and establish the content validity of the framework. The Delphi method was performed under the following principles: 1) including panels involving APN cultivation and potentially employers of APN; 2) incorporating an effective communicating method for the Delphi rounds through the email and web-based survey software; 3) using a structured approach built on previous results in the field; and 4) using adequate rounds to obtain consensus.

### Participants

A purposive stratified sampling method was used to recruit Delphi panels. The 30 panels invited were from different fields, including nursing educators and managers. In the end, a total of 28 experts participated in the study, and two experts were too busy to participate throughout the entire study. All panels were required to be familiar with professional nursing practice and the requirements for the health service workforce, in addition to being credible within their professions.

We selected experts from 15 universities and 13 hospitals in 21 provinces, 4 municipalities and 3 autonomous regions of seven areas to ensure the representativeness of the sample. The inclusion criteria for nursing managers were as follows: (1) had a senior vice title or higher; (2) had worked at least 5 years, including at least 1 year of nursing management experience; and (3) had a bachelor’s degree or above. The inclusion criteria for nursing educators were as follows: (1) the institutions where the experts worked had a master of nursing specialist program (MNS); (2) served as a supervisor of MNS students; and (3) had the title of associate professor or above.

### Instrument

The initial questionnaire of core competencies for APN developed based on the results from Phase 2 included three parts: (1) a cover letter introducing the study and instructing the completion of this questionnaire; (2) core competency framework for APN consisting of six dimensions with 70 items; and (3) experts’ sociodemographic information and their judgement and familiarity with the field of this questionnaire.

### Data collection

The Delphi approaches were conducted from October 2017 to December 2017. We communicated with the Delphi panels through email, and all panelists were kept anonymous throughout the process. For each of our Delphi rounds, panelists were asked to indicate the importance of each domain and the related items within the domains described in the seed statement using a five-point Likert scale with scores ranging from 1 (highly unimportant) to 5 (highly important). Before the rating process, the panels were asked to give comments on the domain delineation. Each question had a blank for the experts to fill in their comments. The experts were asked to complete the questionnaire within 3 weeks. In the next round, panel members were sent the new version of the questionnaire attached to the panel’s comments. Two rounds were finally conducted to reach the final consensus.

### Data analysis

All data were collected using a Web-based online survey and analysis software. The sociodemographic characteristics of the experts were described using appropriate methods. The mean, standard deviation (SD), coefficient of variation (CV), and content validity index (CVI) for each item were calculated. Items were removed if any item met the following criteria simultaneously: mean < 4, CV > 0.25, and CVI < 0.75 [22]; if any item met one or two criteria above, it was kept into the next Delphi round. The authority coefficient (Cr) and respondent rate of experts were also calculated. The authority coefficient was the mean of the sum of the content familiarity coefficient (Cs) and judgement coefficient (Ca), which we collected in the initial questionnaire. The judgement coefficient consisted of four dimensions: working experience, theory analysis, referring to literatures, and self-intuition. The working experience was defined as “great”, “medium” and “small” with a score of 0.5, 0.4 and 0.3 respectively. The theory analysis was defined as “great”, “medium” and “small” with a score of 0.3, 0.2 and 0.1 respectively. The referring to literatures and self-intuition were both defined as “great”, “medium” and “small” with a score of 0.1, 0.1 and 0.05 respectively. And the familiar coefficient ranged from 0.25 to 1.0, indicating “unfamiliar” to “very familiar” [23]. The Cr and respondent rate were acceptable if the value was over 0.7 [24]. Analyses in this phase were conducted in SPSS 23.0.

### Ethical considerations

This study was performed in line with the principles of the Declaration of Helsinki and its later amendments or comparable ethical standards. We got informed consent from all the participants before taking the survey in the study. Approval for the study was granted by the Ethics committee of West China Hospital, Sichuan University (ID: 2017–177).

## Results

### Phase 1: semi-structured interviews with key stakeholders

#### Characteristics of participants

In Phase 1, a total of 22 experts took part in the interviews with a mean age of 47.9 ± 6.7 years old and a mean working time of 27.1 ± 9.1 years from fields of nursing education, management, clinical nursing practice and policy makers; 9 students of nursing specialist programs participated in the interviews with a mean age of 28.4 ± 5.4 years old; 15 nurses with a master of nursing specialist degree took part in the interviews with a mean age of 30.8 ± 3.2 years old and a mean working time of 6.2 ± 4.4 years.

### Competency dimensions

The content analysis summarized six domains of nursing competencies for advanced practice nursing, including direct care practice competency, research competency, professional development competency, organization and management competency, mentoring and consultation competency, and legal/ethical practice (Table 2).

**Table 2:** APNs’ competency domains

Main themes	Categories	Codes/responsibility domain including work tasks
1. Direct care practice competency *(Competency to implement holistic care to patients following nursing procedures to achieve health goals or specific health outcomes.)*	Patient health assessment	Interview Systematic physical examination Observe changes in the patient's condition
	Nursing decision-making	Identify patient’s health problems Assessing the potential and existing risk factors to patients’ health, and actively providing appropriate prevention and treatment measures/health education for disease Interpret, analyze and reach alternative conclusions about patients’ health conditions Utilize medical equipment in an appropriate and accurate manner Undertake Specialized nursing skills safely
	Nurse-patient relationship establishment	Creating external circumstances in patient encounters Creating a safe relationship Counselling of patients and their families.
2. Research competency *(Ability to identify clinical practice problems, to participate in conducting research solutions.)*	Identification research questions	Reflect on own actions Analyze and evaluate own work continuously
	Research implementations	Literature retrieval Design research scheme Executing research project
	Solving clinical problem with Scientific thinking	Reading and applying medical and nursing-related professional literature to clinical care
3.Professional development *(Ability to take active responsibility for one’ own professional development and promoting organization’s professional development)*	Promoting individual development	publishing articles Identify individual development goal Keeps up-to-date knowledge Continually develop own competence
	Promoting professional development	Generate a creative learning environment Develop colleagues’ competence Participates in the development and implementation of health promotion program Share any issue or problem
4.Organization and management *(Ability to collaborates with healthcare team members in order to ensure care quality and patient ‘s safety)*	Multi- professional teamwork	Find solutionsActs as a link between the different professionals Professional support and cooperation between APN
	Safety and risk management	Quality control and management Improving routines Assesses the system risk Application of new techniques Evaluate the effects
5. Mentoring and consultation (*Ability to supervise nursing students, junior nurses and other medical staff, and to provide health education and professional counseling to clients)*	Patients’ health education	Assessing patients’ needs providing information to patients and families Seeking, giving, and receiving help aimed at aiding at patient
	Professional consultation to the service subjects	Consulting Advice giving Right to prescribe medication
	Inter-professional mentoring	Consult other professional experts when required Teaching Guidance Knowledge transmitter Tutor health workers, university students Coach junior nurses
6. Ethical/ legal practice *(Ability to provide nursing care in accordance with laws, regulations, and nursing ethics)*	Legal/Ethical consciousness	Function in accordance with legislative and common law affecting nursing practice Serve as an advocate for the rights of clients
	Legal/Ethical practice	Put emphasis on patients’ own wishes Respect the patient’s right Adopt an ethical approach Carry out nursing practice according to legal requirements and organizational policy

### In phase 2: Delphi Process

After a series of iterations involving five members of our research team, six competency domains encompassing 70 items emerged.

### Sociodemographic data of experts

In Phase 3, a total of 30 experts were initially invited, and 28 (93.3%) experts responded in the first round. The respondent rate in the second round was 100%. Table 3 displays the detailed information of the expert panels. Female experts accounted for the largest proportion (93%), with a mean age of 47.0 ± 6.7 years old. Over half of the experts had worked more than 20 years. Most of the experts served as supervisors for master’s or doctoral students (Table 3).

**Table 3:** Sociodemographic data of the expert panels in the Delphi approach (*N* = 28)

Characteristics	Mean (SD) / N (%)
Age (years)	47.0 ± 6.7
Gender	
Female	26(92.8)
Male	2(7.2)
Education background	
University diploma	3(10.7)
Master’s degree	10(35.7)
Doctoral degree	15(53.6)
Professional title	
Associate professor	11(39.3)
Professor	17(60.7)
Seniority	
10–19 years	11(39.3)
20–29 years	9(32.1)
30–39 years	8(28.6)
Institution	
Hospital	13(46.4)
University/College	15(53.6)
Severed as a supervisor for	
Master	17(60.7)
Doctor	6(21.4)
None of above	5(17.9)
Region	
Northern China	3(10.7)
North eastern China	1(3.6)
Eastern China	5(17.9)
Southern China	3(10.7)
South western China	6(21.3)
North western China	5(17.9)
Central China	5(17.9)

### Reliability of experts

The authority coefficient (Cr), respondent rate, and Kendall’s coefficient of concordance (Kendall’ W) are usually used to evaluate the reliability of a Delphi approach [6, 25]. The content familiarity coefficient (Cs) and judgement coefficient (Ca) were 0.82 and 0.92, respectively. Therefore, the authority coefficient (Cr) was 0.87, which was acceptable. The response rate of both rounds was over 0.9, suggesting good initiative. The values of Kendall’s W were 0.296 and 0.178 for level 1 and level 2 items in the second round (*p* < 0.01), respectively.

### The Core Competencies for APN

**Fig. 1:**
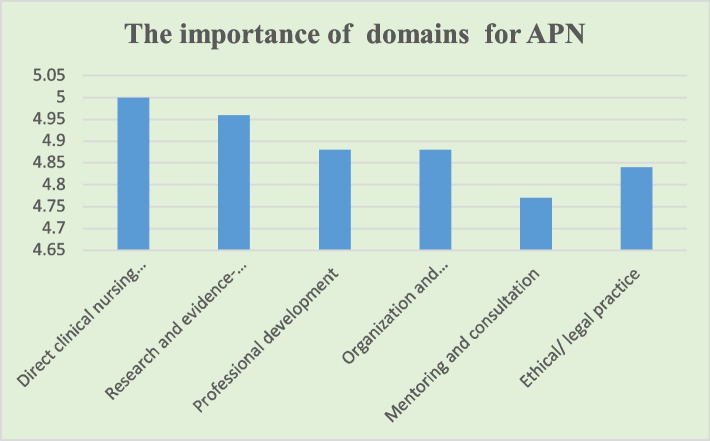
Score on the six dimensions for APN

After two rounds of Delphi approaches, a consensual list of the core competencies for APNs was reached. For the six dimensions, 100% consensus was reached after modifying the description of the dimensions “Direct care practice” and “research” to “Direct *clinical nursing* practice” and “research and *evidence-based nursing* practice” (Table 4). Analysis of the importance score of competency citations by experts revealed that direct clinical nursing practice has more emphasis (Fig. 1).

**Table 4:** Accepted competencies for APN

Items	Mean ± SD	CV	CVI
Direct *clinical nursing* practice *(Ability to implement holistic care to patients following nursing procedures to achieve health goals or specific health outcomes. It includes patient health assessment, clinical nursing decision-making, and nurse-patient relationship establishment in complex clinical situations.)*	5 ± 0	0	1
1.1 The ability to collect disease-related etiological data	4.64 ± 0.73	0.16	1
1.2 The ability to perform systematic physical examination independently	4.75 ± 0.59	0.12	0.95
1.3 The ability to interpret the clinical significance of common auxiliary examination results	4.79 ± 0.42	0.09	1
1.4 The ability to analyse the collected clinical nursing data comprehensively	4.82 ± 0.39	0.08	1
1.5 The ability to make nursing diagnosis for complex, uncertain health problems based on clinical scenarios	4.46 ± 0.64	0.14	0.91
1.6 The ability to develop care plans based on clinical data	4.86 ± 0.36	0.07	1
1.7 The ability to schedule diagnostic and therapeutic prescription drugs rationally	4.61 ± 0.63	0.14	1
1.8 The ability to monitor drug and non-drug treatment within the scope of practice	4.75 ± 0.59	0.12	0.87
1.9 The ability to identify barriers and promoters for treatment plan from patients or family members	4.61 ± 0.69	0.15	0.90
1.10 The ability to adjust nursing plans according to the patient's condition	4.75 ± 0.52	0.11	0.91
1.11 The ability to build therapeutic relationships by facilitating the co-participation of patients and their support systems in the planning, treatment, and management of diseases	4.79 ± 0.50	0.10	0.96
1.12 The ability to observe changes of conditions of common and frequently-occurring diseases in specialized fields and to identify risks in time	4.89 ± 0.31	0.06	1
1.13 The ability to take effective nursing interventions according to changing conditions of patients in time	4.93 ± 0.26	0.05	1
1.14 The ability to identify the priority health problem when facing a clinical emergency	4.93 ± 0.26	0.05	1
1.15 The ability to use common medical equipment skilfully	4.96 ± 0.19	0.04	0.96
1.16 The ability to correctly complete various nursing documents	4.75 ± 0.52	0.11	0.91
1.17 The ability to communicate effectively with critical patients and their families using appropriate communication methods	4.75 ± 0.52	0.11	1
1.18 The ability to provide patients with necessary information including drug efficacy, adverse reactions, and medical costs	4.46 ± 0.64	0.14	1
1.19 The ability to follow up patients in appropriate manners to monitor and evaluate their health/disease status	4.79 ± 0.42	0.09	0.91
1.20 The ability to identify priorities for nursing interventions*	4.75 ± 0.97	0.20	0.95
2. Research and evidence-based nursing practice *(Ability to identify clinical practice problems, conduct research design and finish relevant scientific research independently (implement research protocols and data analysis), and to utilize research findings and apply them to change practice)*	4.96 ± 0.19	0.04	1
2.1 The ability to identify research questions in specialized fields	4.93 ± 0.26	0.05	1
2.2 The ability to search literatures skilfully and use appropriate tools to evaluate literature quality objectively	4.82 ± 0.39	0.08	1
2.3 The ability to design feasible clinical nursing research plans according to research questions	4.71 ± 0.46	0.10	1
2.4 The ability to implement clinical nursing research plans as planned	4.79 ± 0.42	0.09	1
2.5 The ability to draft papers based on scientific research results and promote dissemination and application of results	4.54 ± 0.88	0.19	0.96
2.6 The ability to systematically evaluate the current clinical nursing practice based on latest research results	4.68 ± 0.55	0.12	0.96
2.7 The ability to apply the best evidence to clinical practice	4.86 ± 0.36	0.07	1
2.8 The ability to use effective strategies to improve nursing behaviours and teamwork to promote the adoption of evidence-based nursing practice and innovations	4.71 ± 0.54	0.11	0.91
2.9 The ability to develop or participate in researches focusing on patient care and cost–benefit ratios	4.54 ± 0.58	0.13	0.96
2.10 The ability to monitor the quality of nursing research programs and deal with the problems raising in any research process*	4.75 ± 0.52	0.11	0.95
3. Professional development (*Ability to take active responsibility for one’ own professional development and promoting organization’s professional development. It includes personal and professional development)*	4.88 ± 0.33	0.06	1
3.1 The ability to identify own learning needs	4.82 ± 0.48	0.10	0.96
3.2 The ability to be aware of own strengths and limitations	4.75 ± 0.52	0.11	0.96
3.3 The ability to keep up with advanced developments and new information about the health care system	4.68 ± 0.55	0.12	0.96
3.4 The ability to make personal career development goals	4.89 ± 0.32	0.06	1
3.5 The ability to recognize roles of an individual in the organization and to accomplish tasks of the relevant roles	4.86 ± 0.36	0.07	1
3.6 Have a good physical and mental condition and the ability to adapt to the environment and conduct standardized nursing practice in a constantly changing and evolving healthcare environment	4.82 ± 0.39	0.08	1
3.7 The ability to grasp learning opportunities for personal and professional development to enhance own competences	4.89 ± 0.32	0.06	1
3.8 The ability to identify the role of professional organizations, such as nursing associations, and be actively involved	4.68 ± 0.55	0.12	0.96
3.9 The ability to accept lifelong learning for professional development and maintenance of professional competences	4.61 ± 0.50	0.11	0.96
4. Organization and management (*Ability to collaborates with healthcare team members in order to ensure care quality and patient ‘s safety.it include communication and cooperation effectively with colleagues, and to participate in the organization and coordination of relevant elements of clinical nursing practices to ensure multi-discipline cooperation, nursing quality and patient safety improvement.)*	4.88 ± 0.33	0.06	1
4.1 The ability to establish contacts with academic communities and participate in academic activities	4.54 ± 0.58	0.13	0.96
4.2 The ability to collaborate in the development, implementation, and evaluation of specialized competencies	4.50 ± 0.58	0.12	0.96
4.3 The ability to provide individual, family, and community health services with a multidisciplinary team	4.71 ± 0.54	0.11	0.96
4.4 The ability to serve as a bridge between various medical teams from different fields to coordinate patients’ care and treatment	4.43 ± 0.69	0.16	0.87
4.5 The ability to guide health workers, students, and other persons to acquire new knowledge and skills in a specialized field	4.57 ± 0.57	0.13	0.95
4.6 The ability to coordinate the medical staff of all departments in emergency situations and effectively use team strength to solve problems	4.57 ± 0.57	0.13	0.96
4.7 The ability to foresee the variability of clinical nursing practice and take active intervention measures to ensure the quality of treatment and nursing	4.61 ± 0.63	0.14	0.96
4.8 The ability to skilfully use common quality management tools and analyse the problems existing in nursing quality management	4.750.441	0.09	0.96
4.9 The ability to identify health benefit research questions in nursing practice as a principal investigator or collaborator with a research team	4.710.535	0.11	0.96
5. Mentoring and consultation (*Ability to supervise nursing students, junior nurses and other medical staff, and to provide health education and professional counseling to clients)*	4.77 ± 0.43	0.09	1
5.1 The ability to provide specialized nursing clinics for individuals and their families	4.50 ± 0.92	0.21	0.91
5.2 The ability to provide individuals, families, and communities with knowledge of healthy lifestyles and self-care	4.68 ± 0.55	0.12	1
5.3 The ability to design specialized nursing training programs according to the needs of junior nurses in a specialized field	4.68 ± 0.55	0.12	0.95
5.4 The ability to provide nurses, undergraduates, advanced students with relevant nursing theoretical knowledge and skills, and training of new business and technologies	4.68 ± 0.55	0.12	0.95
5.5 The ability to organize specialized nursing rounds, difficult case discussion, special lectures, etc., and put forward guiding opinions	4.71 ± 0.54	0.11	0.96
5.6 The ability to assess the preferences and available resources of individuals, families, and community residents to facilitate their participation in care decision making and meet their healthcare needs	4.89 ± 0.32	0.06	1
5.7 The ability to provide consultation based on clinical data, theoretical framework, and evidence	4.71 ± 0.54	0.11	0.96
5.8 The ability to provide specialized consultation services for individuals and their families*	4.61 ± 0.57	0.12	0.96
6. Ethical/ legal practice (*(Ability to provide nursing care in accordance with laws, regulations, and nursing ethics)*	4.84 ± 0.37	0.08	1
6.1 The ability to carry out nursing practices in accordance following legal requirements and organizational policies	4.89 ± 0.32	0.06	1
6.2 The ability to consider mental health needs of patients when assessing their health and living conditions	4.86 ± 0.37	0.07	1
6.3 The ability to respect the choice and decision-making rights of patients in nursing practice	4.93 ± 0.26	0.05	1
6.4 The ability to maintain confidentiality and security of medical and nursing data of patents	4.93 ± 0.26	0.05	1
6.5 The ability to respect for the privacy and rights of patients	4.89 ± 0.32	0.06	1

*SD* standard deviation, *CV* coefficient of variation, *CVI* content validity index

*New items proposed by experts

In the “direct clinical nursing practice” dimension, 6 items were modified and merged according to experts’ suggestions; 2 items that did not meet the selection criteria were deleted; and 1 new item proposed by experts was added. In the “research and evidence-based nursing practice” dimension, 3 items were merged, split, or modified, and 3 items that did not meet the item selection criteria were deleted. In the “professional development” dimension, 100% consensus was reached for all items. Regarding the “organization and management” dimension, 4 items that did not meet the item selection criteria were deleted, and 3 items were modified. Regarding the “mentoring and consultation” dimension, 2 items that did not meet the item selection criteria were deleted, and 1 item was split. In the “ethical and legal practice” dimension, only 1 item that did not meet the item selection criteria was deleted. Overall, 6 dimensions with 61 items remained (Table 4).

## Discussion

The core competency framework with six domains and 61 items in this study provided evidence for the role development of APNs, which will prepare a cadre of nurses who have master’s degrees and are consistently ready to face health care delivery challenges and promote health. This study was a first attempt to define the scope of competency for APNs and may accelerate the maturation of the core competency framework for APNs in mainland China.

This study proposed an expert consensual list of the core competencies for APN, which contains six domains: direct clinical nursing practice, research and evidence-based nursing practice, professional development, organization and management, mentoring and consultation, and legal/ethical practice with 61 items. Compared to Hamric’s conceptual model and the ICN’s model, the core competency framework in this study had similar contents but also some differences.

The *direct clinical nursing practice* domain received the highest score among the six domains, as shown in Table 4, which indicated that providing clinical nursing care was the priority of nursing roles (Fig. 1). This finding was consistent with most previous studies [26, 27]. This domain consisted of patient-focused activities, including assessments, clinical nursing decision-making, nurse–patient relationship establishment and interpretation of data, which was consistent with previous studies [28, 29]. Nurse–patient relationships are critical to effectively implement nursing interventions [30]. The ability to establish therapeutic and collaborative nurse–patient relationships was also required in Hamric’s core competency model for APNs [8]. Comments about this domain led to the removal of ‘making a medical diagnosis’, ‘prescribing routine medications and routine examination for the patient’ as an activity, because nurse prescription has not yet been implemented widely in mainland China. If nurse prescription is widely allowed in the future, this activity should be included. Because nurse prescription has been an important responsibility to distinguish general nurses and nurses working in advanced positions in the USA. A total of 20 items were developed to describe the clinical nursing practice competency in detail.

The *research and evidence-based nursing practice competency* ranked second among all domains. This competency was also identified in some previous studies [31–34]. However, neither AMNC nor RCN is reflective of conducting research in their competency framework for APN [11, 12]. AACN only considered translating evidence to practice as a part of the core competencies without emphasizing the research ability of APN [9]. This discrepancy may result from the developing degree of nursing research between Western countries and China. The first master’s degree of nursing programme was not established until 1990 in China [35]. Although the enrolment of master of nursing specialist students has increased gradually since 2010, researchers, policy-makers, and educators think it is critical to have more expectations for the research competency of APNs to improve the nursing discipline in China. In this study, the 10 items in the dimension of research and evidence-based nursing practice pay more attention to the research on clinical problems and the translation of best evidence to clinical practice.

The third identified competency was *professional development*, which included the development of individuals and the nursing discipline. This confirmed previous results from Anna-Lena et al. [28] Krista et al. [27], and Finnbakk et al. [36]. With the development of health technologies, nurses are certainly required to keep up with the leading edge of specialized knowledge and new health care patterns. Moreover, advanced practice nurses are expected to take responsibility for the development of the nursing discipline. However, it may take a long time to make a difference in the acquisition of professional development competency.

Competency in *organization and management* was also required for APNs in this study. This competency contained two aspects: communicating and cooperating effectively with colleagues and participating in the organization and coordination of relevant elements of clinical nursing practice. This finding was slightly different from previous results, which only included collaborative or communicative ability [10, 28, 37] or only included mentoring and quality improvement activities [27, 32, 33]. Previous studies in China have found that communication is a time-consuming activity during the nursing process and directly relates to most conflicts and medical errors in the clinical environment [38, 39]. It was important for APN to conduct effective communication with patients, patients’ family members, and colleagues. The contents of this domain are more similar to professional leadership in Hamric’s advanced practice model.

This study also supported previous results, which stated that *mentoring and consultation* are required competencies for APNs [11, 32, 33, 40]. This competency generally involved health education to patients, professional guidance to peers, and specialized consultation to service subjects in this study, which was a complicated scenario to use clinical knowledge, learning principles, and education tools [41]. This domain covers a broad range of mentoring programs for patients, communities, clinicians, and students. However, the activity within this domain in our research included the large amount of “informal” teaching activities conducted by APN, similar to the result of Chang’s study [24]. It was critical to cultivate the mentoring and consultation competency of APNs to meet the demands of patients about disease management and health resource seeking as well as specialized issues from junior nurses [6].

The *legal/ethical practice* was another core competency identified in this study, which was rarely mentioned in most previous studies. Patient rights protection is a wide consensus around the world, and strengthening the occupational ethics and legal knowledge of nurses was proven to increase service quality and reduce medical disputes [42]. It is urgent to strengthen the ethical and legal practice competency of APNs in the complicated health environments of China [43].

This study identified some similarities with the competence framework of Hamric’s model, namely, six domains. However, there were several differences, such as the domain delineation and some items, due to the cultural and policy differences between China and Western countries. The engagement of stakeholders in all aspects of APN in this study was the distinguishing characteristic of this emerging field. This study also investigated the opinions of a heterogeneous sample, including nurses with master’s degrees, nursing managers and medical policymakers at multiple clinical sites, which helped to make the findings more reliable and generalizable.

### Strengths, limitations and implications

The core competency framework for advanced practice nursing provided a benchmark in the implementation process of new advanced practice nursing roles in mainland China. The findings of this study may provide a framework for curriculum development and performance assessment in master of nursing specialist (MNS) education programe. Future research should focus on embedding these competencies in nursing curricula and how they impact patient outcomes.

This study explored the core competencies for APN in China. We recruited stakeholders including educators, employers, employees, nurses, and policy makers as participants, which provided rich perspectives of this issue. However, the gender of recruited participants disproportionately distributed because of the natural of nursing profession. In addition, in Phase 1, the participants mainly came from west China, which had a relatively lower economic and medical education level. These may affect the results in this study.

## Conclusion

In this study, we identified six core competency domains required for APNs, including direct clinical nursing practice, research and evidence-based nursing practice, professional development, organization and management, mentoring and consultation, and ethical/legal practice. This competency framework can be used in competency-based education to cultivate advanced practice nurses to meet increasing nursing care needs. Additionally, this framework can be used for competency level assessment of APNs. A future study should examine how these competencies are incorporated into nursing curricula and how they affect patient outcomes.

## Supplementary Information

**Additional file 1.**

## Data Availability

The data that support the findings of this study are available from the corresponding author upon reasonable request. The data are not publicly available due to privacy or ethical restrictions.
