# Supplementary material for: Developing a core competency framework for advanced practice nursing in mainland China: a sequential exploratory study
Source: BMC Nurs. 2023 May 23;22:179. doi: 10.1186/s12912-023-01335-4 (PMC10207664; doi:10.1186/s12912-023-01335-4)
Supplement: Supplementary file 1 — Additional file 1. [file 12912_2023_1335_MOESM1_ESM.docx]

Table S1 The Value of Judgment Coefficient

| Criterion | Influence degree | | |
| --- | --- | --- | --- |
|  | Great | Medium | Small |
| Working experience  Theory analysis  Referring to literatures  Self-intuition | 0.5  0.3  0.1  0.1 | 0.4  0.2  0.1  0.1 | 0.3  0.1  0.05  0.05 |

Table S2 The Degree of Familiarity with Content

| Familiarity Degree | Very Familiar | | Familiar | General | Unfamiliar |
| --- | --- | --- | --- | --- | --- |
| Self-rated score | | 1 | 0.75 | 0.5 | 0.25 |

Table S3 Socio-demographic characteristics of experts

| ID | Gender | Age | Education background | Woking years | Professional title/position | Working field |
| --- | --- | --- | --- | --- | --- | --- |
| E1  E2  E3  E4  E5  E6  E7  E8  E9  E10  E11  E12  E13  E14  E15  E16  E17  E18  E19  E20  E21  E22 | Female  Female  Female  Female  Female  Female  Female  Female  Female  Female  Female  Female  Male  Female  Female  Female  Female  Female  Female  Female  Male  Male | 51  38  52  42  47  48  46  48  41  48  50  52  36  52  51  34  50  49  66  51  50  52 | Master’s degree  Master’s degree  University diploma  Master’s degree  Master’s degree  Master’s degree  Master’s degree  Doctoral degree  Master’s degree  Master’s degree  University diploma  University diploma  Master’s degree  University diploma  University diploma  Master’s degree  Master’s degree  Doctoral degree  Technical college diploma  Doctoral degree  University diploma  University diploma | 35  6  34  24  17  28  28  30  20  28  30  34  12  33  32  11  33  30  46  32  25  27 | Professor  Associate professor  Associate professor  Associate professor  Professor  Professor  Professor  Professor  Professor  Professor  Professor  Professor  Professor  Associate professor  Associate professor  Nursing Supervisor  Professor  Professor  Professor  Professor  Section chief  Section chief | Nursing education  Nursing education  Nursing practice  Nursing education  Nursing education  Nursing practice  Nursing practice  Nursing education  Nursing education  Nursing management  Nursing practice  Nursing practice  Psychiatric nursing  Surgical nursing  Medical nursing  Paediatric nursing  Nursing management  Gynaecological Nursing  Nursing management  Nursing education  Hospital management  Academic certification |

Table S4 Socio-demographic characteristics of graduate students of MNS

| ID | Gender | Age | Having working experience | Having Nurse Practitioner Certificate |
| --- | --- | --- | --- | --- |
| G1  G2  G3  G4  G5  G6  G7  G8  G9 | Female  Male  Female  Female  Female  Female  Female  Female  Female | 26  26  33  23  25  24  25  39  35 | Yes  Yes  No  Yes  Yes  Yes  Yes  No  No | Yes  Yes  Yes  Yes  Yes  Yes  Yes  Yes  Yes |

MNS: masters of nursing specialist

Table S5 Socio-demographic characteristics of nurses with a master of nursing specialist degree

| ID | Gender | Age | Working years | Time after getting master’s degree | Professional title | Department |
| --- | --- | --- | --- | --- | --- | --- |
| P1  P2  P3  P4  P5  P6  P7  P8  P9  P10  P11  P12  P13  P14  P15 | Female  Female  Female  Female  Male  Female  Female  Male  Male  Female  Female  Female  Male  Female  Male | 30  29  36  33  27  36  31  35  32  33  26  37  29  28  30 | 4  3  11  8  2  10  3  7  3  8  1  8  6  2  7 | 4  3  11  8  2  10  3  5  2  8  1  8  1  2  2 | Intermediate  Intermediate  Intermediate  Intermediate  Senior nurse  Intermediate  Intermediate  Intermediate  Intermediate  Intermediate  Senior nurse  Associate professor  Intermediate  Senior nurse  Intermediate | Medical department  Medical department  Gynaecological department  Medical department  Department of Nursing  Paediatric department  Gynaecological department  Oncology department  Operating room  Emergency department  Out-patient department  Surgical department  Surgical department  Psychiatric department  Psychiatric department |
